# Prevalence of *PALB2* Mutations in Breast Cancer Patients in Multi-Ethnic Asian Population in Malaysia and Singapore

**DOI:** 10.1371/journal.pone.0073638

**Published:** 2013-08-20

**Authors:** Sze Yee Phuah, Sheau Yee Lee, Peter Kang, In Nee Kang, Sook-Yee Yoon, Meow Keong Thong, Mikael Hartman, Jen-Hwei Sng, Cheng Har Yip, Nur Aishah Mohd Taib, Soo-Hwang Teo

**Affiliations:** 1 Cancer Research Initiatives Foundation, Sime Darby Medical Centre, Subang Jaya, Selangor, Malaysia; 2 Breast Cancer Research Unit, University Malaya Cancer Research Institute, Faculty of Medicine, University Malaya Medical Centre, University Malaya, Kuala Lumpur, Malaysia; 3 Department of Paediatrics, Faculty of Medicine, University Malaya Medical Centre, Kuala Lumpur, Malaysia; 4 Saw Swee Hock School of Public Health, National University of Singapore and National University Health System, Singapore, Singapore; 5 Department of Surgery, National University of Singapore and National University Health System, Singapore, Singapore; 6 Department of Surgery, Yong Loo Lin School of Medicine, National University of Singapore, Singapore, Singapore; 7 Sime Darby Medical Centre, Subang Jaya, Selangor, Malaysia; 8 Department of Surgery, Faculty of Medicine, University Malaya Medical Centre, Kuala Lumpur, Malaysia; IFOM, Fondazione Istituto FIRC di Oncologia Molecolare, Italy

## Abstract

**Background:**

The partner and localizer of breast cancer 2 (PALB2) is responsible for facilitating BRCA2-mediated DNA repair by serving as a bridging molecule, acting as the physical and functional link between the breast cancer 1 (BRCA1) and breast cancer 2 (BRCA2) proteins. Truncating mutations in the *PALB2* gene are rare but are thought to be associated with increased risks of developing breast cancer in various populations.

**Methods:**

We evaluated the contribution of *PALB2* germline mutations in 122 Asian women with breast cancer, all of whom had significant family history of breast and other cancers. Further screening for nine *PALB2* mutations was conducted in 874 Malaysian and 532 Singaporean breast cancer patients, and in 1342 unaffected Malaysian and 541 unaffected Singaporean women.

**Results:**

By analyzing the entire coding region of *PALB2*, we found two novel truncating mutations and ten missense mutations in families tested negative for *BRCA1/2*-mutations. One additional novel truncating *PALB2* mutation was identified in one patient through genotyping analysis. Our results indicate a low prevalence of deleterious *PALB2* mutations and a specific mutation profile within the Malaysian and Singaporean populations.

## Introduction

PALB2 (PArtner and Localizer of BRCA2) is a protein that interacts with BRCA2, stabilizing the intranuclear accumulation of BRCA2 proteins at sites of DNA damage [1]. Biallelic loss of PALB2 causes increased predisposition to cancers, increased sensitivity to DNA damaging agents and Fanconi’s Anemia [2].

Germline *PALB2* mutations are rare, but have been reportedly associated with increased predisposition to breast and other cancers. In multiple-breast cancer case families, germline *PALB2* mutations have been reported in 1.1% (10 out of 920), 2.7% (3 out of 113), 2.0% (1 out of 50), and 0.6% (5 out of 779) of Western European families in the United Kingdom, Finland, French-Canada, and Australasian, respectively [3–6]. Within the familial context, germline *PALB2* mutations are associated with a 2.3 to 6 fold increased risk to breast cancer [3,7,8]. Germline *PALB2* mutations have also been reported in unselected female breast cancer cases, but at a lower prevalence (0.9%, 18 out of 1,918 in Finnish breast cancer cases [4]; 0.5%, 2 out of 356 in French-Canadian breast cancer patients aged <50 years old [5]; and 0.4%, 5 out of 1,403 in Australian breast cancer patients [6]). Notably, deleterious germline *PALB2* mutations have also been reported in pancreatic cancer cohorts from United States [9] and Europe [10], but its association with increased risk to ovarian cancer cohorts remains controversial [11,12].

Germline *PALB2* mutations have also been described in other populations. In a study of Han Chinese, two carriers were identified out of 360 (0.6%) high risk breast cancer patients analysed [13]. Notably, three mutations (1.1%) were reported in a study of *PALB2* mutations in 279 African-American patients [14]. Taken together, these data suggests that mutations in *PALB2* are rare and may be population-specific.

In this study, we have analysed the prevalence of *PALB2* mutations in a cohort of 122 familial breast cancer cases who have previously been tested negative for germline mutations in *BRCA1* and *BRCA2* and determined the prevalence of selected mutations in a further unselected cohort of breast cancer cases and controls from Malaysia and Singapore.

## Materials and Methods

### Ethics statement

All study subjects were provided written informed consent. Blood, demographic and family history data was collected from breast cancer patients who consented to participate in this study. The study was approved by the Medical Ethics Committee of University Malaya Medical Centre (UMMC).

### Study subjects

#### (a) Familial breast cancer patients for DNA sequencing

The recruitment of breast cancer patients into the Malaysian Breast Cancer Genetic Study (MyBrCa) started in January 2003 at the University Malaya Medical Centre in Kuala Lumpur. All were histopathology-proven breast carcinoma. From January 2003 to December 2010, a total of 1,220 breast cancer patients were recruited into the MyBrCa study. All index patients were asked about family history of any cancer, including all third degree relatives. Mutation detection for germline *BRCA1* and *BRCA2* mutations was conducted on 402 individuals by direct DNA sequencing and multiple ligation dependent probe amplification (MLPA) as previously described [15,16]. Of these, 155 individuals were included in the PALB2 study because they were either (a) diagnosed ≤50 years old and had at least one first- or second-degree relatives with breast cancer; (b) diagnosed with bilateral breast cancer with the primary cancer at age 50 or below; (c) diagnosed with male breast cancer at any age; (d) affected with both breast and ovarian cancers; (e) affected with both breast and pancreatic cancers; or (f) diagnosed with breast cancer and had at least one first-degree relative with pancreatic cancer.

#### (b) Malaysian breast cancer cases and controls for genotyping

All women with invasive breast cancer who participated in the MyBrCa study between January 2003 and December 2010 were included in hospital-based cohort of breast cancer cases. Of the 1,220 breast cancer patients, 195 individuals were excluded from the study if they were either (a) diagnosed with non-invasive carcinoma (51 cases); (b) had missing histopathology data (74 cases); (c) from mixed parentage or other ethnicities (20 cases); and (d) had insufficient or low quality genomic DNA samples (52 cases). Of the remaining individuals, a total of 874 breast cancer cases consisting of 528 (out of 621) Chinese, 180 (out of 224) Malay and 1166 (out of 180) Indian cases were included in the study.

Women with no breast cancer were selected from two sources. The first was a cohort of 270 healthy individuals attending the hospital for non-cancer related ailments and who had no family history of cancer in first or second degree relatives (270 healthy individuals comprising 90 Chinese, 90 Malays and 90 Indians). The second cohort comprised 1,072 women attending an opportunistic mammographic screening programme from October 2011 to September 2012.

#### (c) Singaporean breast cancer cases and controls for genotyping

An additional 532 breast cancer cases from Singapore Breast Cancer Cohort Project and 541 age-matched healthy controls from the Multi-ethnic Cohort in Singapore Consortium of Cohort Studies (SCCS) were analysed in this study. The recruitment of Singapore Breast Cancer Cohort Project started in April 2010 at National University Hospital and included both prevalent and incident breast cancer cases diagnosed from 1988 onwards. 740 patients have been recruited between April 2010 and March 2011 and 75% of total participants donated blood or saliva. Cases were excluded from current analysis due to (a) from mixed parentage or other ethnicities (13 cases), (b) male gender (1 case) and (c) had insufficient or low quality of genomic DNA samples (7 cases). The Multi-ethnic Cohort in SCCS consisted of approximately 12,000 community-dwelling individuals who are Singaporeans or Singaporean Permanent Residents, 21 years and older. Exclusion criteria were a medical history of cancer, acute myocardial infarction or stroke, or major psychiatric morbidity including schizophrenia, psychotic depression, and advanced Alzheimer’s Disease. 77% of the participants who were interviewed provided a blood sample.

### PALB2 analysis

Sequencing of all the intron-exon junctions and exonic sequences was conducted using Sanger sequencing of genomic DNA extracted from peripheral white blood cells using standard methods. Primer sequences were adapted from several publications (Table S1) [2,6,17].

### PALB2 Genotyping

Multiplex genotyping of seven PALB2 mutations identified in exon 4 in this study and an additional two Asian recurrent mutations were performed using high-throughput Sequenom MassARRAY iPLEX platform (Sequenom Inc., San Diego, California, USA). Mutations in other exons were identified after the completion of the genotyping study and were therefore excluded from analyses. All mutations identified were confirmed by direct sequencing in an independent DNA sample.

### In silico analysis

In silico analysis of the effect of missense mutations on protein function was determined using Polyphen-2 (Polymorphism Phenotyping version 2) [18] and SIFT (Sorting Tolerant from Intolerant) [19,20].

## Results

### Prevalence of PALB2 germline mutations in familial breast cancer patients

Of the 155 high risk breast cancer patients selected for this study, 20 had germline deleterious mutations in *BRCA1* and 13 in *BRCA2*. The remaining 122 individuals tested negative for *BRCA* mutations were analyzed for *PALB2* germline mutations by direct Sanger sequencing (Table 1) and 13 genetic variants were identified (Table 2). These included two deleterious mutations (mutation prevalence of 1.6%), ten missense variants of unknown clinical significance and one synonymous variant.

**Table 1:** Characteristics of 122 individuals tested for *PALB2* germline mutations.

**Characteristics**	**No**	**%**
**Ethnicity**		
Chinese	82	67.2
Malay	25	20.5
Indian	12	9.8
Others	3	2.5
**Female breast cancer**	121	99.2
**Male breast cancer**	1	0.8
**Age at diagnosis**		
≤ 35	28	23.0
36-40	31	25.4
41-50	55	45.1
51-60	5	4.1
> 60	3	2.5
**Personal cancers**		
Bilateral cancer:		
1 ≤ 50 years	21	17.2
Ovarian cancer	6	4.9
Pancreatic cancer	1	0.8
**Manchester Score**		
< 15	83	68.0
≥ 15	39	32.0
**Family history of cancers (up to 3rd degree)**		
Breast cancer	100	82.0
Ovarian cancer	3	2.5
Pancreatic cancer	2	1.6
Prostate cancer	3	2.5
**No family history of either breast, ovarian, pancreatic and prostate cancers**	20	16.4

In total, 122 breast cancer patients who were tested negative for germline mutations in *BRCA1* and *BRCA2* were analyzed for germline mutations in *PALB2* by Sanger sequencing. Table 1 shows the distribution of index patients according to their ethnicity, age at diagnosis and family history characteristics.

**Table 2:** List of 13 *PALB2* genetic variants detected.

Exon	Type	Mutation/Variant			Cases (n=122)		Ethnicity
		Exonic	HGVS	AA change	Frequency	%	
4	Deletion	1237_1241 delAAGAA	c.1037_1041 delAAGAA	STOP 358	1	0.8	Chinese
7	Deletion	2806 delC	c.2606 delC	S869X	1	0.8	Chinese
4	Missense	946 C>T	c.746 C>T	P249L	1	0.8	Chinese
4	Missense	1099 C>T	c.899 C>T	T300I	1	0.8	Indian
4	Missense	1125 A>G	c.925 A>G	I309V	1	0.8	Chinese
4	Missense	1254 G>C	c.1054 G>C	E352Q	1	0.8	Chinese
4	Missense	1692 G>T	c.1492 G>T	D498Y	1	0.8	Chinese
4	Missense	1859 C>A	c.1659 A>G	H553Q	1	0.8	Chinese
4	Missense	1876 A>G	c.1676 A>G	Q559R	38	31.1	28 Chinese, 6 Malays, 3 Indians and 1 others
5	Missense	2489 G>C	c.2289 G>C	L763F	1	0.8	Malay
10	Missense	3249 G>A	c.3049 G>A	A1017T	1	0.8	Malay
10	Missense	3254 G>C	c.3054 G>C	E1018D	1	0.8	Chinese
5	Synonymous	2267 G>A	c.2067 G>A	S689S	1	0.8	Chinese

Table 2 shows all 13 *PALB2* germline mutations detected from the sequencing of 122 *BRCA*-negative individuals and their respective mutation prevalence in the study.

#### (a) Mutation c.1037_1041 delAAGAA; STOP 358

**Figure 1:**
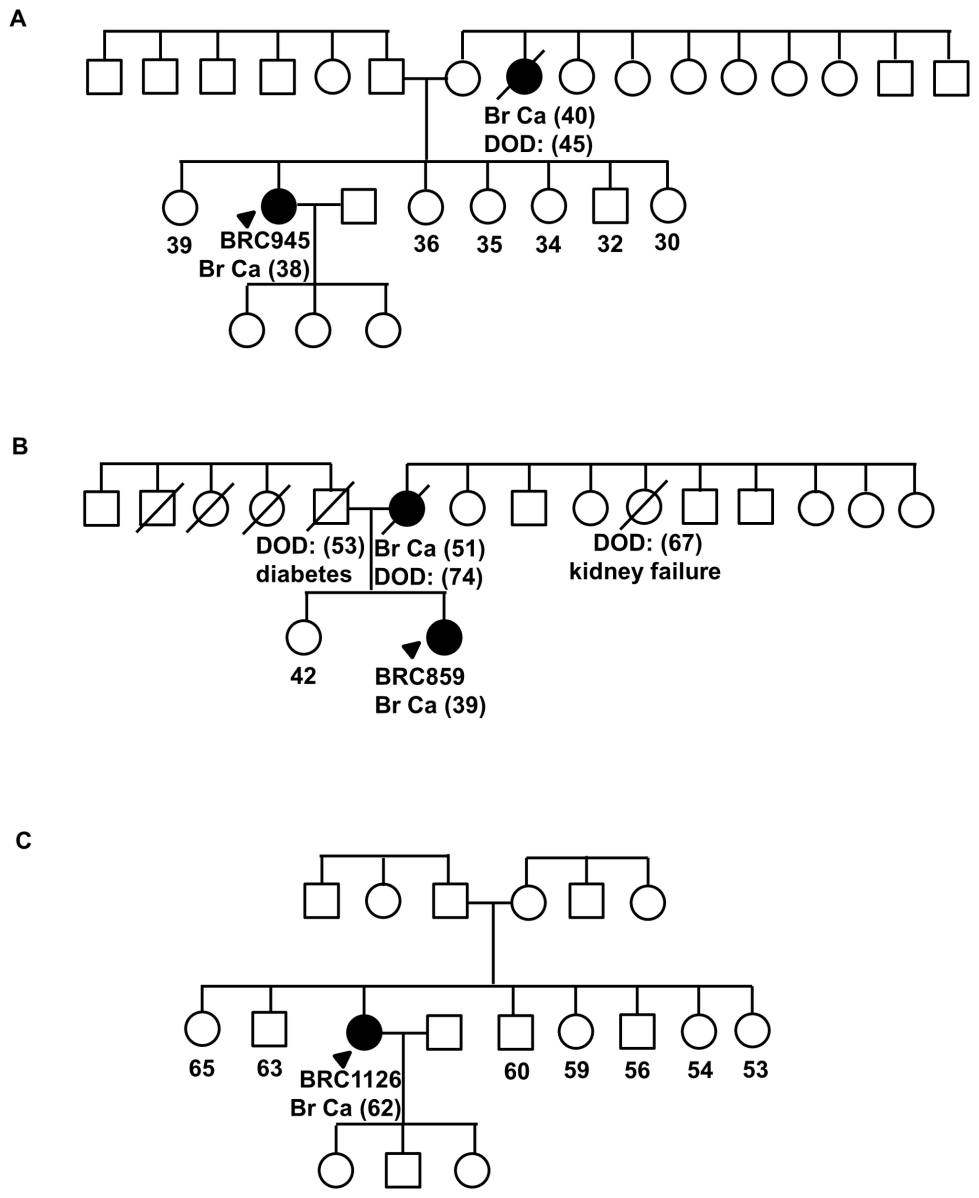
Pedigrees of families with germline mutations in *PALB2*. (A) Family pedigree of BRC945, carrier of c.1037_1041delAAGAA [STOP358]. (B) Family pedigree of BRC859, carrier of c.2606delC [S869X]. (C) Family pedigree of BRC1126, carrier of c.1050delAACA [STOP353]. Index patients are indicated with an arrow while individuals affected with breast cancer are indicated with filled symbol. Date of birth (DOB) and age of diagnosis (in bracket) foraffected individuals are indicated. Deceased individuals are indicated with a slash.

This truncating mutation was found in a Chinese woman who developed invasive ductal carcinoma at age 38, which was negative for the expression of the estrogen- and HER-2 receptors and positive for the expression of the progesterone-receptor. She had a maternal aunt who had breast cancer in her 40s (Figure 1 A).

#### (b) Mutation c.2606 delC; S869X

This truncating mutation was found in a Chinese woman who developed invasive ductal carcinoma at age 39, which was positive for the expression of both estrogen- and progesterone-receptors and had amplification of HER-2 receptor. Her mother was affected with breast cancer at age 51 (Figure 1 B).

### Evaluation of variants pathogenicity through case-control analysis

Rare mutations detected from the sequencing of exon 4 of *PALB2* (n=7) and deleterious mutations that were reported to be recurrent in other Asian populations (Chinese *PALB2* c.751 C>T and *PALB2* c.1250_1251 delAAinsTCT) [13] were tested for in additional cohorts of breast cancer cases and controls by genotyping (Table 3). The cases comprised a hospital-based cohort of 874 breast cancer cases from Malaysia and 532 breast cancer cases from Singapore. The unaffected controls comprised 270 women attending non-cancer related procedures at the same hospital, 1072 women attending mammogram screening and 541 population-based controls from Singapore.

**Table 3:** Case-control analysis of nine *PALB2* variants identified from exon 4 sequencing (n=7) and Asian populations (n=2).

Exon	Type	Mutation/Variant			Malaysia Case-control						Singapore Case-Control			
		Exonic	HGVS	AA change	Cases (874)	(%)	Controls (270)	(%)	Controls (1072)	(%)	Cases (532)	(%)	Controls (541)	(%)
4	Deletion	1237_1241 delAAGAA	c.1037_1041 delAAGAA	STOP 358	1/868	0.1	0/264	0.0	0/1058	0.0	0/519	0.0	0/536	0.0
4	Missense	1876 A>G	c.1676 A>G	Q559R	286/871	32.8	70/257	27.2	na	na	na	na	na	na
4	Missense	1125 A>G	c.925 A>G	I309V	7/870	0.8	2/270	0.7	16/1079	1.5	14/507	2.8	13/519	2.5
4	Missense	1692 G>T	c.1492 G>T	D498Y	5/845	0.6	1/225	0.4	13/1060	1.2	5/526	1.0	10/528	1.9
4	Missense	946 C>T	c.746 C>T	P249L	1/872	0.1	1/270	0.4	0/1002	0.0	0/529	0.0	0/536	0.0
4	Missense	1254 G>C	c.1054 G>C	E352Q	1/870	0.1	0/262	0.0	0/1079	0.0	0/527	0.0	1/535	0.2
4	Missense	1859 C>A	c.1659 A>G	H553Q	1/870	0.1	0/269	0.0	0/1078	0.0	1/514	0.2	0/528	0.0
4	Chinese deletion	951 C>T	c.751 C>T	Q251X	0/870	0.0	0/265	0.0	0/1051	0.0	0/523	0.0	0/536	0.0
4	Chinese deletion	1250_1251 delAAinsTCT	c.1050_1051 delAAinsTCT	STOP 353	1/873	0.1	0/267	0.0	0/1059	0.0	0/519	0.0	0/538	0.0

#### (a) Deleterious mutations

One of the three deleterious mutations evaluated was detected in the Malaysian breast cancer cases and none were identified in the Malaysian unaffected women, Singaporean breast cancer cases or unaffected women (Table 3). Sequence analysis after genotyping also revealed a novel *PALB2* frameshift mutation, *PALB2* c.1050 delAACA occurring at the same amino acid as the tested mutation, *PALB2* c.1050 delAAinsTCT.

The patient with this novel deleterious mutation was a Chinese woman who developed infiltrating ductal carcinoma at age 62, which was positive for the expression of the estrogen receptor and negative for the expression of the progesterone- and HER2 receptors. Her paternal uncle was diagnosed with nasopharyngeal cancer in his 70s (Figure 1 C).

#### (b) Missense variants

*PALB2* Q559R was determined to be common in the cases and controls (33% and 27% respectively) and this variant was also found in five homozygote unaffected controls (data not shown) showing that this variant is a polymorphism.

Both *PALB2* I309V and *PALB2* D498Y variants were detected in more than 1% of unaffected women from the Malaysian and Singaporean control cohorts suggesting that these are polymorphisms. Notably, *PALB2* I309V was predicted to be benign based on SIFT and Polyphen-2 in-silico predictions whereas *PALB2* D498Y was suggested to be possibly damaging in these in-silico analysis.

The remaining three missense mutations were determined to be rare. *PALB2* H553Q, predicted to be benign by Polyphen-2 and SIFT, was found in one Malaysian and one Singaporean breast cancer cases (0.1% and 0.2% respectively) and none in the controls. *PALB2* P249L and *PALB2* E352Q were each found in one breast cancer case and one control. In-silico predictions suggest that P249L may be benign while E352Q is probably damaging.

Four other missense mutations (*PALB2* T300I, L763F, A1017T and E1018D) were not tested in the case-control analysis. Variants pathogenicity prediction using in-silico tools suggest that *PALB2* T300I is benign while Polyphen-2 predicted *PALB2* L763F, *PALB2* A1017T and *PALB2* E1018D to be probably damaging.

## Discussion

In this study, we have analysed the prevalence of *PALB2* mutations in a cohort of 122 familial breast cancer cases who do not have alterations in *BRCA1* or *BRCA2* and found 2 (1.3%) were *PALB2* carriers. Notably, we did not examine large chromosomal rearrangements which have previously been reported in other studies [21,22] and it is possible that these rare genetic changes could have account for additional *PALB2* carriers in our population. Although the patients in this study comprised 3 ethnic groups (Chinese, Malay and Indian), all 3 individuals with *PALB2* deleterious mutations are Chinese. There was only 4 missense variants found in the Malay and Indian women in this study (compared to 7 in the Chinese women) and this is likely to be because the majority of the women in this study are Chinese. The rarity of germline *PALB2* mutations is consistent with that reported in other populations where a mutation prevalence of 0-2% has been reported [17,23–25].

Our study also identified 10 *PALB2* missense variants, of which 5 are novel [25,26]. Of these, 6 were tested in a case-control analysis and our results suggest that 5 are likely to be either benign (*PALB2* Q559R, I309V, D498Y, P249L and E352Q) or associated with a low increased risk of breast cancer. *PALB2* H553Q was found in 2 women affected with breast cancer but not in 1,875 controls. Further analysis of this and other variants [T300I, L763F, A1017T, E1018D] require other methods, including in vitro functional characterization [27] and co-segregation analyses.

Our study uncovered an unusual situation where genotyping of a deleterious mutation (*PALB2* c.1050 delAAinsTCT) [13] revealed a distinct mutation (*PALB2* c.1050 delAACA) at the same site. Sequence alteration caused by deletion of AACA resulted in mutant SNP that is similar to the interrogated SNP and was therefore regarded as a positive screen. This highlights the limitation of genotyping assay in distinguishing distinct mutations which share a common mutant SNP and therefore, confirmation by conventional sequencing is necessary.

Notably, of the 3 individuals with germline deleterious *PALB2* mutations, 2 have moderate family history with Manchester score of 12 and 14 respectively, whereas one developed breast cancer at late age in the absence of any family history of cancer (Manchester score of 2). Indeed, by contrast to *BRCA1* and *BRCA2* mutation status, we found that there was no association between *PALB2* mutation status and family history of breast, ovarian, prostate or pancreatic cancer (data not shown). This is consistent with other studies that have also shown no familial clustering of breast cancer in *PALB2* families compared to non-BRCA carrier families [3,14,23]. The family members of the *PALB2* carrier families were not available for co-segregation analysis and therefore it was not possible to determine the increased risk caused by germline mutation of *PALB2* in our population.

In summary, we found 2 germline *PALB2* deleterious mutation carriers in 122 high risk non-*BRCA1* or *BRCA2* breast cancer patients by DNA sequencing and 1 germline carrier in 1406 breast cancer patients by genotyping. Our data shows that *PALB2* germline mutations are rare, and are associated with family history of breast cancer in some, but not all families.

## Supporting Information

Table S1 **Amplification primers used for the sequencing of PALB2 gene.**
(DOCX)

## References

[1] Xia B, Sheng Q, Nakanishi K, Ohashi A, Wu J et al. (2006) Control of BRCA2 cellular and clinical functions by a nuclear partner, PALB2. Mol Cell 22: 719-729. doi:10.1016/j.molcel.2006.05.022. PubMed: 16793542. 16793542 10.1016/j.molcel.2006.05.022

[2] Reid S, Schindler D, Hanenberg H, Barker K, Hanks S et al. (2007) Biallelic mutations in PALB2 cause Fanconi anemia subtype FA-N and predispose to childhood cancer. Nat Genet 39: 162-164. doi:10.1038/ng1947. PubMed: 17200671. 17200671 10.1038/ng1947

[3] Rahman N, Seal S, Thompson D, Kelly P, Renwick A et al. (2007) PALB2, which encodes a BRCA2-interacting protein, is a breast cancer susceptibility gene. Nat Genet 39: 165-167. doi:10.1038/ng1959. PubMed: 17200668. 17200668 10.1038/ng1959 PMC2871593

[4] Erkko H, Xia B, Nikkilä J, Schleutker J, Syrjäkoski K et al. (2007) A recurrent mutation in PALB2 in Finnish cancer families. Nature 446: 316-319. doi:10.1038/nature05609. PubMed: 17287723. 17287723 10.1038/nature05609

[5] Foulkes WD, Ghadirian P, Akbari MR, Hamel N, Giroux S et al. (2007) Identification of a novel truncating PALB2 mutation and analysis of its contribution to early-onset breast cancer in French-Canadian women. Breast Cancer Res 9: R83. doi:10.1186/bcr1828. PubMed: 18053174. 18053174 10.1186/bcr1828 PMC2246183

[6] Southey MC, Teo ZL, Dowty JG, Odefrey FA, Park DJ et al. (2010) A PALB2 mutation associated with high risk of breast cancer. Breast Cancer Res 12: R109. doi:10.1186/bcr2796. PubMed: 21182766. 21182766 10.1186/bcr2796 PMC3046454

[7] Hollestelle A, Wasielewski M, Martens JW, Schutte M (2010) Discovering moderate-risk breast cancer susceptibility genes. Curr Opin Genet Dev 20: 268-276. doi:10.1016/j.gde.2010.02.009. PubMed: 20346647. 20346647 10.1016/j.gde.2010.02.009

[8] Erkko H, Dowty JG, Nikkilä J, Syrjäkoski K, Mannermaa A et al. (2008) Penetrance analysis of the PALB2 c.1592delT founder mutation. Clin Cancer Res 14: 4667-4671. doi:10.1158/1078-0432.CCR-08-0210. PubMed: 18628482. 18628482 10.1158/1078-0432.CCR-08-0210

[9] Hofstatter EW, Domchek SM, Miron A, Garber J, Wang M et al. (2011) PALB2 mutations in familial breast and pancreatic cancer. Fam Cancer 10: 225-231. doi:10.1007/s10689-011-9426-1. PubMed: 21365267. 21365267 10.1007/s10689-011-9426-1 PMC3836668

[10] Slater EP, Langer P, Niemczyk E, Strauch K, Butler J et al. (2010) PALB2 mutations in European familial pancreatic cancer families. Clin Genet 78: 490-494. doi:10.1111/j.1399-0004.2010.01425.x. PubMed: 20412113. 20412113 10.1111/j.1399-0004.2010.01425.x

[11] Prokofyeva D, Bogdanova N, Bermisheva M, Zinnatullina G, Hillemanns P et al. (2012) Rare occurrence of PALB2 mutations in ovarian cancer patients from the Volga-Ural region. Clin Genet 82: 100-101. doi:10.1111/j.1399-0004.2011.01824.x. PubMed: 22310028. 22310028 10.1111/j.1399-0004.2011.01824.x

[12] Dansonka-Mieszkowska A, Kluska A, Moes J, Dabrowska M, Nowakowska D et al. (2010) A novel germline PALB2 deletion in Polish breast and ovarian cancer patients. BMC Med Genet 11: 20. doi:10.1186/1471-2156-11-20. PubMed: 20122277. 20122277 10.1186/1471-2350-11-20 PMC2829009

[13] Cao AY, Huang J, Hu Z, Li WF, Ma ZL et al. (2009) The prevalence of PALB2 germline mutations in BRCA1/BRCA2 negative Chinese women with early onset breast cancer or affected relatives. Breast Cancer Res Treat 114: 457-462. doi:10.1007/s10549-008-0036-z. PubMed: 18446436. 18446436 10.1007/s10549-008-0036-z

[14] Zheng Y, Zhang J, Niu Q, Huo D, Olopade OI (2012) Novel germline PALB2 truncating mutations in African American breast cancer patients. Cancer 118: 1362-1370. doi:10.1002/cncr.26388. PubMed: 21932393. 21932393 10.1002/cncr.26388 PMC3244533

[15] Thirthagiri E, Lee SY, Kang P, Lee DS, Toh GT et al. (2008) Evaluation of BRCA1 and BRCA2 mutations and risk-prediction models in a typical Asian country (Malaysia) with a relatively low incidence of breast cancer. Breast Cancer Res 10: R59. doi:10.1186/bcr1943. PubMed: 18627636. 18627636 10.1186/bcr2118 PMC2575532

[16] Toh GT, Kang P, Lee SS, Lee DS, Lee SY et al. (2008) BRCA1 and BRCA2 germline mutations in Malaysian women with early-onset breast cancer without a family history. PLOS ONE 3: e2024. doi:10.1371/journal.pone.0002024. PubMed: 18431501. 18431501 10.1371/journal.pone.0002024 PMC2295262

[17] Tischkowitz M, Xia B, Sabbaghian N, Reis-Filho JS, Hamel N et al. (2007) Analysis of PALB2/FANCN-associated breast cancer families. Proc Natl Acad Sci U S A 104: 6788-6793. doi:10.1073/pnas.0701724104. PubMed: 17420451. 17420451 10.1073/pnas.0701724104 PMC1871863

[18] Adzhubei IA, Schmidt S, Peshkin L, Ramensky VE, Gerasimova A et al. (2010) A method and server for predicting damaging missense mutations. Nat Methods 7: 248-249. doi:10.1038/nmeth0410-248. PubMed: 20354512. 20354512 10.1038/nmeth0410-248 PMC2855889

[19] Kumar P, Henikoff S, Ng PC (2009) Predicting the effects of coding non-synonymous variants on protein function using the SIFT algorithm. Nat Protoc 4: 1073-1081. doi:10.1038/nprot.2009.86. PubMed: 19561590. 19561590 10.1038/nprot.2009.86

[20] Ng PC, Henikoff S (2003) SIFT: Predicting amino acid changes that affect protein function. Nucleic Acids Res 31: 3812-3814. doi:10.1093/nar/gkg509. PubMed: 12824425. 12824425 10.1093/nar/gkg509 PMC168916

[21] Blanco A, de la Hoya M, Balmaña J, Ramón y Cajal T, Teulé A et al. (2012) Detection of a large rearrangement in PALB2 in Spanish breast cancer families with male breast cancer. Breast Cancer Res Treat 132: 307-315. doi:10.1007/s10549-011-1842-2. PubMed: 22052327. 22052327 10.1007/s10549-011-1842-2

[22] Xia B, Dorsman JC, Ameziane N, de Vries Y, Rooimans MA et al. (2007) Fanconi anemia is associated with a defect in the BRCA2 partner PALB2. Nat Genet 39: 159-161. doi:10.1038/ng1942. PubMed: 17200672. 17200672 10.1038/ng1942

[23] Sluiter M, Mew S, van Rensburg EJ (2009) PALB2 sequence variants in young South African breast cancer patients. Fam Cancer 8: 347-353. doi:10.1007/s10689-009-9241-0. PubMed: 19333784. 19333784 10.1007/s10689-009-9241-0

[24] García MJ, Fernández V, Osorio A, Barroso A, Llort G et al. (2009) Analysis of FANCB and FANCN/PALB2 fanconi anemia genes in BRCA1/2-negative Spanish breast cancer families. Breast Cancer Res Treat 113: 545-551. doi:10.1007/s10549-008-9945-0. PubMed: 18302019. 18302019 10.1007/s10549-008-9945-0

[25] Ding YC, Steele L, Chu LH, Kelley K, Davis H et al. (2011) Germline mutations in PALB2 in African-American breast cancer cases. Breast Cancer Res Treat 126: 227-230. doi:10.1007/s10549-010-1271-7. PubMed: 21113654. 21113654 10.1007/s10549-010-1271-7 PMC3457798

[26] Haiman CA, Han Y, Feng Y, Xia L, Hsu C et al. (2013) Genome-wide testing of putative functional exonic variants in relationship with breast and prostate cancer risk in a multiethnic population. PLOS Genet 9: e1003419. 23555315 10.1371/journal.pgen.1003419 PMC3610631

[27] Vallon-Christersson J, Cayanan C, Haraldsson K, Loman N, Bergthorsson JT et al. (2001) Functional analysis of BRCA1 C-terminal missense mutations identified in breast and ovarian cancer families. Hum Mol Genet 10: 353-360. doi:10.1093/hmg/10.4.353. PubMed: 11157798. 11157798 10.1093/hmg/10.4.353 PMC4756649

