# Supplementary material for: Prevalence of PALB2 Mutations in Breast Cancer Patients in Multi-Ethnic Asian Population in Malaysia and Singapore
Source: PLoS One. 2013 Aug 20;8(8):e73638. doi: 10.1371/journal.pone.0073638 (PMC3748013; doi:10.1371/journal.pone.0073638)
Supplement: Table S1 — Amplification primers used for the sequencing of PALB2 gene. (DOCX) [file pone.0073638.s001.docx]

**Table S1: Amplification primers used for the sequencing of *PALB2* gene**

| **Exon** | **F/R** | **Primer** | **Primer Sequence** | **Length (bp)** | **Tm**  **(°C)** |
| --- | --- | --- | --- | --- | --- |
| 1 | F | ex 1-F | GGATTTAATTGGCCGGAGTT | 309 | 60°C |
|  | R | ex 1-R | GACACAAAGCCAGGCCTAAA |  |  |
| 2,3 | F | ex2,3_T_F | GTGCTACTCCCTGCCTCTTG | 471 | 60°C |
|  | R | ex 3-R | CACACTGTGGGAAAAAGAACAA |  |  |
| 4Ha | F | ex 4Ha-F | GCCTGAATGAAATGTCACTGATT | 495 | 60°C |
|  | R | ex 4Ha-R | GGTGATCTAGCAGGATTTTTGC |  |  |
| 4Hb | F | ex 4Hb-F | CCCTAGTGGTGAGCAAAAGC | 388 | 56°C |
|  | R | ex 4Hb-R | TTCAAGGTGCTGACTACTACCG |  |  |
| 4Sb | F | ex 4Sb-F | GCAAAAATCCTGCTAGATCACC | 556 | 56°C |
|  | R | ex 4Sb-R | AGATTTTCATTCCTGCCATCA |  |  |
| 4Sc | F | ex 4Sc-F | CCAGCAAATGAAAACCAAAA | 396 | 56°C |
|  | R | ex 4Sc-R | GCATGTGCCAGACATCCTAA |  |  |
| 4Sd | F | ex 4Sd-F | GAGTCATTTGGATGTCAAGAAAAA | 470 | 56°C |
|  | R | ex 4Sd-R | AAGGAAGTGCCAGGCAAATA |  |  |
| 5A | F | ex 5A-F | TTGTCTGTTTTGTTGGGTTTTG | 563 | 60°C |
|  | R | ex 5C-R | TAAGATGGGGAAAGCAGGTG |  |  |
| 5B | F | ex 5D-F | TTGCGCCTGATGATAATGAC | 462 | 60°C |
|  | R | ex 5E3-R | GCAAGCAAGTCATGCTGTTTA |  |  |
| 6 | F | ex 6-F | AGTGGGTAATGCAGGCAGAC | 213 | 60°C |
|  | R | ex 6-R | TGACTGAATTCTTTTCAGTTCATT |  |  |
| 7 | F | ex 7-F | TGCTTTGCATAAAACAGCACT | 293 | 60°C |
|  | R | ex 7-R | TGGTAAGCTGCCCATCTACA |  |  |
| 8 | F | ex 8-F | TGGAAAATCTGGATTAAACAAAAA | 221 | 58°C |
|  | R | ex 8-R | TGCACTTAAAACCAGCTGACA |  |  |
| 9 | F | ex 9A-F | ATTAAAAGGTTACTCCTCACATCAC | 346 | 60°C |
|  | R | ex 9Seq-R | TGTTGATGCGGTACATGCTT |  |  |
| 10 | F | ex 10-F | CCTAGAGACTGCTTTAGTGCAAA | 250 | 58°C |
|  | R | ex 10-R | TTCACAACAACCCTGTAAAATTAG |  |  |
| 11 | F | ex 11A-F | TTTTCTGAATACTGGTTTGTTGGA | 203 | 58°C |
|  | R | ex 11B-R | CACTTAATGAGACCAACAGTAACACA |  |  |
| 12 | F | ex 12A1-F | GAGCCTATCGGTCATTGCTT | 305 | 58°C |
|  | R | ex 12B-R | TTTCAGAATGTCCCACCCATAGA |  |  |
| 13 | F | ex13A1-F | TCTTCTTTGTATGCTATCAGGTTCCT | 578 | 60°C |
|  | R | ex13_T-R | TGCTTCTGCAAATGATCTTGA |  |  |
